# Non-adherence to self-care practices & medication and health related quality of life among patients with type 2 diabetes: a cross-sectional study

**DOI:** 10.1186/1471-2458-14-431

**Published:** 2014-05-07

**Authors:** Farzana Saleh, Shirin J Mumu, Ferdous Ara, Md Abdul Hafez, Liaquat Ali

**Affiliations:** 1 Department of Community Nutrition, Bangladesh University of Health Sciences (BUHS), 125/1 Darussalam Mirpur-1, Dhaka-1216, Bangladesh; 2 Department of Epidemiology, Bangladesh University of Health Sciences (BUHS), Dhaka, Bangladesh; 3 Department of Biostatistics, Bangladesh University of Health Sciences (BUHS), Dhaka, Bangladesh; 4 Department of Biochemistry and Cell Biology, Bangladesh University of Health Sciences (BUHS), Dhaka, Bangladesh

**Keywords:** Non-adherence, Self-care practices, Medication, Health related quality of life, Bangladesh, Type 2 diabetes

## Abstract

**Background:**

Non-adherence to lifestyle modification among diabetic patients develops the short-term risks and the long-term complications as well as declines the quality of life. This study aimed to find out the association between non-adherence to self-care practices, medication and health related quality of life (HR-QoL) among type 2 diabetic patients.

**Methods:**

At least 1 year diagnosed patients with type 2 diabetes (N = 500), age>25 years were conveniently selected from the Out-Patient Department of Bangladesh Institute of Health Sciences Hospital. Patients’ self-care practices were assessed via interviewer-administered questionnaires using an analytical cross-sectional design. HRQoL was assessed by an adapted and validated Bangla version of the EQ-5D (EuroQol Group, 2009) questionnaire which has five domains- mobility, self-care, usual activities, pain/discomfort and anxiety/depression and two levels on each dimension. EQ-5D responses were further translated into single summery EQ-5D index using UK TTO value set. Patients’ were considered as non-adhered to self-care practices according to the guidelines of Diabetic Association of Bangladesh. Multivariable linear regression was used to assess the association between non-adherence towards self-care practices and HRQoL.

**Results:**

Among the study patients, 50.2% were females and mean ± SD age was 54.2 (±11.2) years. Non-adherence rate were assessed for: blood glucose monitoring (37%), diet (44.8%), foot care (43.2%), exercise (33.2%) and smoking (37.2%). About 50.4% patients had problem in mobility, 28.2% in self-care, 47.6% in usual activities, 72.8% in pain/discomfort and 73.6% in anxiety/depression. On chi-squared test, significant association was found between non adherence to foot care and problem with mobility, self-care and usual activities (p < 0.05). Significant association was also found between non-adherence to exercise and poor mobility, self- care, usual activities, pain and anxiety (p < 0.05). Non-adherence to diet was associated with poor mobility (p < 0.05). In multivariable linear regression non-adherence to foot care (p = 0.0001), exercise (p = 0.0001), and smoking (p = 0.047) showed significant association with EQ-5D index after adjusting co-variates.

**Conclusions:**

In this study, patients who have a non-adherence rate also have a lower quality of life.

## Background

In the 21st century, we have seen more globalization and industrialization, longer life spans and changes in lifestyles worldwide. A consequence of these changes will be shifts in the patterns of disease, with chronic diseases such as diabetes becoming more prevalent [1]. International Diabetes Federation (IDF) 2013 projects the prevalence of diabetes in Bangladeshi is 7.11% [2]. Due to the large number of people involved and its associated complications, the disease warrants urgent attention. The cost of diabetes care is huge (total US$ 142; US$ 88 to patient and US$ 54 to provider) in developing countries like Bangladesh [3]. Apart from the cost of treatment, diabetes also affects the quality of life both in the patient and the people around them because of its chronic nature and multiorgan involvement.

It is clear that chronically ill individuals had lower mean health related quality of life (HR-QoL) domain scores when compared to healthy adults [4]. Thus, type 2 diabetics have to face many problems which may an impact on their HR-QoL. At the same time the success of long-term maintenance therapy and good metabolic control depends largely upon the patient’s adherence and behavior in terms of keeping appointments, taking medication and making lifestyle changes.

The definition of adherence, according to the World Health Organization, is the extent to which a persons’ behavior – taking medication, following a diet, and/or performing lifestyle changes – corresponds with agreed recommendations from the health care provider [5]. In our previous study, the rate of non-adherence was in diet (88%); in exercise (25%); in blood glucose testing (32%); in foot care (70%); in smoking (6%) and in betel quid chewing habit (25%) [6].

The management regimen [blood glucose (BG) monitoring, diet, physical activity, foot care and medication] that is associated with bringing diabetes under control can reduce diabetes-related morbidity and mortality and simultaneously increase patients’ HR-QoL.

Different studies showed the association between non-adherence to self-care practices, treatment, medication and HR-QoL [7-9]; one study showed the efficacy of the intervention program in improving quality of life among diabetic patients [10].

Individuals with the disease have to make major lifestyle changes and learn to live with monitoring BG, using multiple drugs and injections, and dealing with treatment and complications of the disease. In every aspect the primary goal in the management of chronic disease is the improvement of the patient’s HR-QoL.

As with the increasing number of diabetic patients more and more will need specialized diabetic care, education and motivation and failure to provide above these they will develop more complications, increase treatment cost and reduce their quality of life. There have been few studies on non-adherence to self-care practices and health related quality of life among type 2 diabetic patients in developing countries like Bangladesh and the objective of this study was to find out the association between non-adherence to self-care practices, medication and health related quality of life among type 2 diabetic patients.

## Methods

An analytical cross-sectional study design was adopted, and 500 type 2 diabetic patients were selected conveniently in consideration of the inclusion and exclusion criteria from an outpatient department (OPD) setting at Bangladesh Institute of Health Sciences (BIHS) hospital.

The minimum required sample size was calculated using the formula n = Z^2^ pq/d^2^ (Where, z = 1.96, p = the expected non-adherence proportion towards medication, i.e., 52.1% [9], q = 1-p, and d = allowable error of known prevalence, i.e., 5%). Patients who were > 25 years and diagnosed for at least 1 year were included. Patients who had other medical complications or were unable to answer a short list of simple questions (sociodemographic information such as name, address, disease complications, etc.) were excluded from the study.

A three-part questionnaire was designed by the researcher. The first part of the questionnaire consisted of socio-demographic information and family history of diabetes. Part two contained self-care practices related to diabetes (BG monitoring, diet, foot care, exercise, smoking and medication) and part three focused on the questionnaire consisted of an adapted and validated Bangla (local language) version of the EQ-5D (EuroQol Group, 2009) [11]. EQ-5D questionnaire has five domains- mobility, self-care, usual activities, pain/discomfort and anxiety/depression and two levels on each dimension. The developers of the EQ-5D have generated value sets in several countries to calculate a preference-based index for the 243 health states defined by responses to the 5 questions of the EQ-5D, using a scale on which 0.0 represents being dead and 1.0 full health. Values of the index can be negative for states that are deemed to be “worse than death”: so for example, the minimum value in the UK based value set [12] is -0.59 which represents the worst possible health state (i.e., 33333). As there is no value set based on time trade off developed for the South-East Asian population we have used the time trade off (TTO) method conducted in the United Kingdom [12] i.e., UK TTO most commonly used sets currently available for the EQ-5D.

Anthropometric measurements, clinical and biochemical reports were collected from patients’ diabetes guide book by using checklist. Checklist means an instrument used when observing some situation. The researcher/interviewers put tick marks against the particular point or write down what he/she observes.

According to the guidelines of Diabetic Association of Bangladesh (BADAS) [13], two-point scale (adherence and non-adherence) were used to assess patients’ non-adherence to BG monitoring, diet, foot care, exercise, smoking and medication.

The health care providers write subsequent date of blood testing on patient guide book in relation to the patients’ physiological condition and patients were considered non-adhered if they missed or did not perform BG testing on prescribed date.

For diet, patients were non- adhered if they did not follow the recommended dietary chart, not maintain specific time of food intake and did not follow advised quantity and quality of food by using three days food diary.

Exercise, foot care, smoking and medication were measured by taking history of the patients. Patients accepted non-adhered in exercise if they did exercise < 45 min/day.

Non-adhered towards foot care if they did not follow the basic foot care principles, such as, daily examination, cleaning, using moisturizer, wearing correct size and shape shoe and cutting nail.

Regarding smoking, patients were considered as non-adhered if they did not quit smoking after getting advice from health care provider.

Non-adhered to medication if one of the following answers was positive from among the possible choices: I don’t observe the time of the intake, I change the prescribed amount or dose of the medicine, I take more than the prescribed amount, I change the prescribed amount or dose of the medicine, I take less than the prescribed amount, I change the time of the intake of the medicine and the amount as well. In addition, cross-check was done by showing their prescription.

A pre-test was conducted before the questionnaire was finalized. Statistical tests were considered significant at p-values ≤ 5% (≤ 0.05). EQ-5D responses were further translated into single summery EQ-5D index using UK TTO value set. Frequencies were calculated for descriptive analysis. Chi-squared tests were performed on categorical data to find the relationships between variables. Multivariable linear regression was used to assess the association between non-adherence towards self-care practices and HRQoL.

Informed written consent was obtained from all respondents after a full explanation of the nature, purpose, and procedures used for the study. Ethical approval was obtained from the ethics and research review committees of the Diabetic Association of Bangladesh.

## Results

Mean age of the patients was 54.2 ± 11.2 years and 92.2% patients belonged to middle to upper-middle age group; half (50.2%) of the patients were female. About 41% had completed high school and 50.8% came from lower-middle income family. Half (52.2%) of the patients attended diabetes education class at least once, 43.8% had never attend. Mean BMI was 26.1 (±6.7) kg/m^2^ and about 78.8% were overweight or obese according to Asian BMI cut-off value [14].

**Figure 1:**
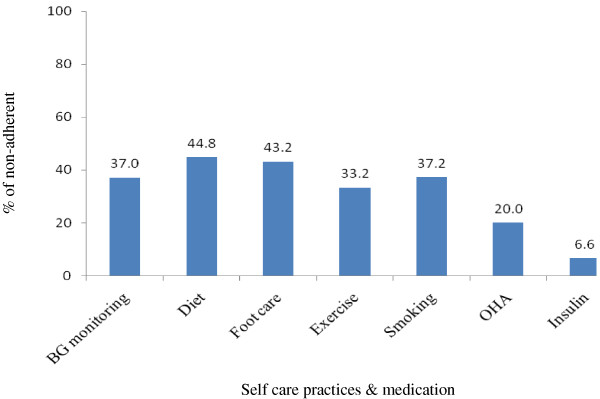
Distribution of the patients according to non-adherence to their self-care practices & medication.

The non-adherence rate among the patients is shown in Figure 1. Around 37% did not adhere to BG monitoring. Non-adherence to diet was 44.8%, to foot care 43.2% and to exercise 33.2%. About 37.2% were non-adherent to smoking after receiving advice. Non-adherence to OHA was 20% while 6.6% non-adhered to insulin.

**Figure 2:**
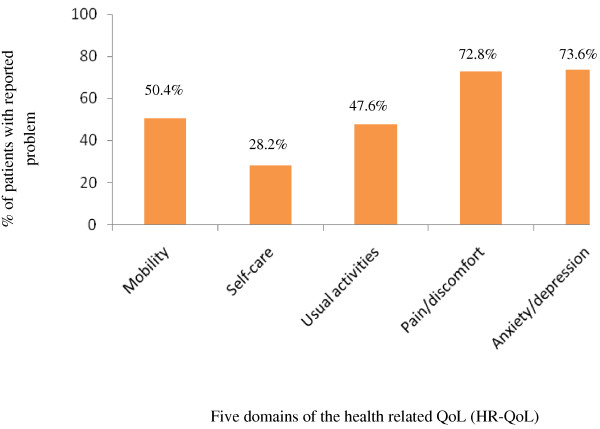
Profile of the health related quality of life (HR-QoL) among the patients.

About 50.4% patients had problem in mobility, 28.2% in self-care, 47.6% in usual activities, 72.8% in pain/discomfort and 73.6% in anxiety/depression (Figure 2).

Table 1 shows the relationship between the levels of non-adherence to self-care practices & medication Vs HR-QoL among the patients. A significant relationship existed between the non-adherence to diet and with poor mobility (p = 0.041) and 54.9% had problem in mobility. Those who were non-adhere to foot care had problem in mobility (57.9%), usual activities (54.2%) and the association was significant (p ≤ 0.05) but the exception was found in self care, only 37% had problem and the relationship was significant (p ≤ 0.05). Non-adherence to exercise and problem with mobility (65.5%), self care (42.3%), usual activities (62.5%), pain/discomfort (80.4%) and anxiety/depression (82.1%) was also found a significant (p ≤ 0.05) association.

**Table 1:** Relationship between the level of non-adherence to self-care practices & medication Vs health related quality of life (N = 500)

**HR-QoL**	**Non-adherence to**						
	**BG monitoring (%)**	**Diet (%)**	**Foot care (%)**	**Exercise (%)**	**Smoking (%)**	**Taking OHA (%)**	**Taking insulin (%)**
Mobility							
No problem	50.8	45.1	42.1	34.5	50	47	43.8
Problem	49.2	54.9	57.9	65.5	50	53	56.3
*X*^2^/p	0.172/0.711	4.284/0.041	8.48/0.004	23.004/0.0001	0.349/0.752	0.02/0.91	0.029/1.000
Self-care							
No problem	73.5	71	63	57.7	87.5	65	62.5
Problem	26.5	29	37	42.3	12.5	35	37.5
*X*^2^/p	0.426/0.538	0.784/0.419	14.66/0.0001	24.71/0.0001	0.267/0.695	2.06/0.16	0.814/0.404
Usual activities							
No problem	55.1	50	45.8	37.5	75	49	31.3
Problem	44.9	50	54.2	62.5	25	51	68.8
*X*^2^/p	0.881/0.355	1.58/0.228	6.57/0.011	22.52/0.0001	2.252/0.199	0.26/0.65	4.14/0.05
Pain/discomfort							
No problem	29.2	22.3	24.1	19.6	31.3	24	21.9
Problem	70.8	77.7	75.9	80.4	68.8	76	78.1
*X*^2^/p	0.587/0.467	0.049/0.885	1.87/0.188	7.29/0.008	0.02/1.000	0.67/0.44	0.285/0.664
Anxiety/depression							
No problem	25.4	21.9	28.7	17.9	18.8	23	15.6
Problem	74.6	78.1	71.3	82.1	81.3	77	84.4
*X*^2^/p	0.150/0.753	0.443/0.566	1.03/0.35	9.5/0.002	0.29/0.719	0.54/0.52	3.42/0.08

Results were expressed as %; Fisher’s exact test was conducted when cells have expected count < 5; *p ≤ 0.05 was used as the limit for significance.

To identify the factors that might predict the patients’ quality of life, multivariable linear regression analyses were performed. All the six parameters viz., non-adherence to BG monitoring, to dietary practice, to foot care, to exercise, to smoking, to OHA and to insulin were included simultaneously in the regression model. Three viz., non-adherence to foot care, to exercise and to smoking shown significant association with EQ-5D index; three individual models were fitted separately for each of these significant parameters. The result shows that the overall multiple regression model that was used to assess predictions of EQ-5D index with non-adherence to foot care achieved an R^2^ of 0.23; p = 0.0001; non-adherence to foot care was significantly associated with the EQ-5D index after adjustment (p = 0.0001; 95% CI: 0.054 to 0.166) (Table 2).

**Table 2:** Multivariable regression analysis of EQ-5D index value as a dependent variable with non-adherence to foot care of the respondents (N = 500)

***Predictor variable***	***B***^***1***^ ***± SE***	***Beta***^***2***^	***P value***	***95% CI for B***	
				***Lower***	***Upper***
Age	-0.006 ± 0.001	-0.194	0.0001	-0.009	-0.003
Gender	-0.283 ± 0.032	-0.404	0.0001	-0.347	-0.220
Habitat	0.021 ± 0.033	0.027	0.518	-0.044	0.087
Prescribed treatment	-0.029 ± 0.010	-0.111	0.006	-0.049	-0.008
Education	0.035 ± 0.015	0.102	0.023	0.005	0.065
Occupation	-0.008 ± 0.016	-0.021	0.636	-0.039	0.024
Duration of DM	-0.004 ± 0.002	-0.082	0.055	-0.007	0.0001
Number of education class	-0.031 ± 0.024	-0.050	0.205	-0.079	0.017
Family history of DM	0.033 ± 0.017	0.083	0.045	0.001	0.066
Monthly income	-0.010 ± 0.022	-0.018	0.651	-0.053	0.033
Non-adherence to foot care	0.110 ± 0.29	0.156	0.0001	0.054	0.166

[1 = Unstandardized sample regression co- efficient, 2 = Standardized sample regression co- efficient].

Adjusted R^2^ = 23%; Overall model F-test, p = 0.0001.

Predictions of EQ-5D index with non-adherence to exercise achieved an R^2^ of 0.25; p = 0.0001; respondents who were non-adherent to exercise had a significant association (p = 0.0001; 95% CI: 0.102 to 0.218) with the quality of life (Table 3).

**Table 3:** Multivariable regression analysis of EQ-5D index value as a dependent variable with non-adherence to exercise of the respondents (N = 500)

***Predictor variable***	***B***^***1***^ ***± SE***	***Beta***^***2***^	***P value***	***95% CI for B***	
				***Lower***	***Upper***
Age	-0.005 ± 0.001	-0.151	0.001	-0.008	-0.002
Gender	-0.251 ± 0.032	-0.359	0.0001	-0.314	-0.188
Habitat	0.012 ± 0.033	0.015	0.710	-0.052	0.076
Prescribed treatment	-0.024 ± 0.010	-0.092	0.019	-0.044	-0.004
Education	0.044 ± 0.015	0.130	0.003	0.015	0.074
Occupation	-0.009 ± 0.016	-0.026	0.554	-0.040	0.022
Duration of DM	-0.004 ± 0.002	-0.087	0.038	-0.007	0.0001
Number of education class	-0.039 ± 0.024	-0.062	0.110	-0.086	0.009
Family history of DM	0.040 ± 0.016	0.101	0.014	0.008	0.072
Monthly income	-0.018 ± 0.022	-0.033	0.408	-0.060	0.025
Non-adherence to exercise	0.160 ± 0.030	0.216	0.0001	0.102	0.218

[1 = Unstandardized sample regression co- efficient, 2 = Standardized sample regression co- efficient].

Adjusted R^2^ = 25%; Overall model F-test, p = 0.0001.

In regression analysis also found significant predictions of respondents’ EQ-5D index with non-adherence to smoking (R^2^ = 0.21; p = 0.0001). Non-adherence to smoking was significantly associated with the EQ-5D index after adjustment (p = 0.047; 95% CI: 0.0001 to 0.0001) (Table 4).

**Table 4:** Multivariable regression analysis of EQ-5D index value as a dependent variable with non-adherence to smoking of the respondents (N = 500)

***Predictor variable***	***B***^***1***^ ***± SE***	***Beta***^***2***^	***P value***	***95% CI for B***	
				***Lower***	***Upper***
Age	-0.006 ± 0.001	-0.187	0.0001	-0.009	-0.003
Gender	-0.299 ± 0.034	-0.427	0.0001	-0.367	0.231
Habitat	0.015 ± 0.034	0.019	0.654	-0.051	0.081
Prescribed treatment	-0.025 ± 0.010	-0.097	0.017	-0.045	-0.005
Education	0.039 ± 0.015	0.113	0.013	0.008	0.069
Occupation	-0.012 ± 0.016	-0.035	0.445	-0.044	.020
Duration of DM	-0.003 ± 0.002	-0.076	0.076	-0.007	0.0001
Number of education class	-0.037 ± 0.025	-0.060	0.130	-0.086	0.011
Family history of DM	0.036 ± 0.017	0.092	0.030	0.004	0.069
Monthly income	0.0001 ± 0.022	-0.001	0.979	0.044	0.043
Non-adherence to smoking	0.0001 ± 0.0001	0.086	0.047	0.0001	0.0001

[1 = Unstandardized sample regression co- efficient, 2 = Standardized sample regression co- efficient].

Adjusted R^2^ = 21%; Overall model F-test, p = 0.0001.

## Discussion

The main goal of diabetes management is to improve the quality of life of the patients so that they can possibly have normal life. Successful management depends upon the extent to which a person’s adherence of keeping appointments, monitoring his/her glycemic status, taking medication and making lifestyle changes. Few studies regarding the non-adherence to self-care practices and HR-QoL among type 2 diabetic patients are available in Bangladesh or elsewhere in the world. This study was undertaken in order to assess the association between non-adherence to self-care practices, medication and health related quality of life among type 2 diabetic patients.

In our study, the rate of non-adherence to BG monitoring was 37% and was consistent with our previous study (32%) [6]. In Mexican study, only 17.2% of patients showed good treatment adherence behavior [7]; in Iran [8], 93.7% and in Hungary [9] 80% patients did not follow the guideline and perform their blood glucose monitoring. Thus the numbers of our non-adherent patient may be lower compared to others but not optimum level and this is due to the patient’s lack of time, gives less importance, distance, financial problem and feels not uncomfortable without checking glucose.

Important therapies in the management of diabetes are an adjustment of diet, foot care, doing exercise and cessation of smoking.

Almost every patient in BIHS hospital received instructions on the most appropriate diet from their health care providers. These instructions referred to a limited carbohydrate-intake diet. However, the patients did not pay any attention to them, habitually feel sick after taking prescribed amount and in some case they have lack of facilities to follow the instructions. Near about half of the patients (45%) were non-adhered with their prescribed diet in the present study; the previous non-adherence rate was 88% [6]. Other studies showed the non-adherence to diet of 60%–80% [8,9]. In India, the occurrence of non-adherence to diet was 50% which is comparable to our present study [15]. Probably the same culture and lifestyle are responsible for this.

Ulceration and amputation of the lower extremities are among the most serious complications of diabetes. In the study four of ten diabetic patients did not administer foot care and 70% non-adhered to foot care in our previous study [6] though our present result is better than previous one but higher than Iran study (20%) [8]. The reasons are that most of the elderly patients have memory problems and decreased cognitive function, and often health care providers do not give emphasis on foot care.

Exercise is another important part of managing diabetes because it improves insulin action, reduce weight, decrease glucose intolerance and the occurrence of complications. Two-thirds of the patients in the study were non-adhered in doing their physical activity in spite of the importance of exercise. In our previous study, non-adherence rate for exercise was 25% [6]; in Iran the rate was 75% [8]; in Hungary it was 67% [9] and in India the rate was 42.9% [15]. This may be reflected the gradual changes in lifestyle among our patents. Now they understand that daily activities are not considered as exercise though still there are some barriers to do the regular physical activities, such as, busy schedule, family problem, not aware about the benefits and the difference in the occurrence of non-adherence might be due to different patient co-morbidities such as hypertension, obesity, and osteoarthritis.

In the present study, we found that patients did not keep to the instructions of the health care provider regarding cessation of smoking. After receiving advice, 37.2% diabetic patients did not adhere to smoking. In Hungary, it was 14.8% which was comparatively average with their general population [9]. On the other hand, in India 32.4% were smoker [15] and in Health Insurance Organization (HIO) in Alexandria after diagnosis 27.6% continued to smoke as before [16].

Another vital part in the management of diabetes is the use of medications. Patients should be motivated to use the medications prescribed. The rate of non-adherence to the intake of OHA and Insulin was 20% and 6.6% in the study respectively. In Iran, it was found that 35% of diabetics always and 65% of them often non-adhered to treatment regimen [8]. Moreover, in Hungary, non-adherence to the intake of the drug(s) was found 50% [9]. Though the adherence rate is comparatively better in our study but some common reasons played vital role in the occurrence of non-adherence to medications in all populations, such as, patients forget to take their medicines, active rejection of therapy, expensive medication, large number of medicines to be taken simultaneously, lack of health improvement, side-effects and absence of signs of illness.

Counseling and education play a key role in the improvement of the people’s quality of life. In German study [10], patients who participating in the disease management program (DMP) for type 2 diabetes mellitus showed significantly higher ratings of their HR-QoL in the dimensions mobility, self-care and performing usual activities compared to patients in routine care (RC). In our study, only half (50%) of the patients attended diabetes education class at least once, except self-care, majority (70%) of the patients had problem in pain/discomfort and anxiety/depression and 50% faced problem with mobility and usual activities.

The non-adherence parameters had an unenthusiastic effect on quality-of-life domains. Significant association was found between non-adherence to diet, foot care and exercise with poor mobility, self-care, usual activities, pain/discomfort and anxiety/depression. More than half (54.9%) of diabetic patients with poor mobility had significantly non-adherent to take food in time and in appropriate amount. Similar results were found in association between non-adherence to foot care and problem with mobility, self-care, usual activities and pain/discomfort. The only exception was 75.4% patients with anxiety showed adherence to take care of their feet than patients with non-adherence group but not significantly. In case of non-adherence to exercise and five domains of HR-QoL, a significant relationship was found and more than half of the patients who showed non-adherence in doing exercise had problem with five domains.

HRQoL among the patients is generally low. The mean rated score for EQ-5D index in this study was 0.55. The index value (EQ-5D index) was significantly associated with non-adherence to foot care, duration of exercise, and smoking but no significant relationship was found between BG monitoring, dietary practice, medication and HR-QoL. Our results also indicate that the elevated occurrence of non-adherence is associated with low quality of life. Therefore, the probable reasons of non-adherence are increasing duration of disease, patients might be fed up with the treatment regimen to follow; in elderly have decreased cognitive function and complications. However, no association between quality of life and treatment adherence behavior was found in Mexican study [7].

In the present study, in managing diabetes may be there was a gap between what the patients should do and what they were actually doing. Therefore, the patients’ level of effort was not uniform in all areas of managing their disease. Effective and cultural oriented education intervention successfully enhances adherence and simultaneously increases the quality of life and also vice versa.

The limitations of the present study were the cross-sectional design discovered the quality of life and adherence at the same time. In this study patients were self-reported and the results might not be giving the true reflection in all aspects. Besides this, variables were continuous and they changed over time, measurements at only one point in time as taken here had more limited value. Patients were enrolled by using convenient sampling technique so that the results could not be represented the whole diabetic patients of Bangladesh.

## Conclusions

In the present study, we may conclude that patients who have a non-adherence rate also have a lower quality of life. It is recommended that every effort be made to initiate and to promote act change and to improve quality of life in people with diabetes. To achieve this, an appropriate, strong and effective patient education, motivation program and patient-doctor relationship should be planned. The present data could be used in developing fruitful intervention program for our patients and in improving their quality of life. A longitudinal study would also provide a more complete picture regarding which of these variables could predict the others.

## Competing interests

The authors declare that they have no competing interests.

## Authors’ contributions

FS: contributed her intellectual ability to conception and design of the research, analysis and interpretation of data, drafting the article, revising it critically for important intellectual content, and final approval of the version to be published; SJM: contributed her intellectual ability to conception and design of the research, analysis and interpretation of data, revising it critically for important intellectual content; FA: contributed her intellectual ability to conception and design of the research, analysis and interpretation of data, revising it critically for important intellectual content; MAH: analysis and interpretation of data, revision of manuscript for important intellectual content; LA: contributed his intellectual ability to conception and design of the research. All of the above authors read and approved the final manuscript.

## Pre-publication history

The pre-publication history for this paper can be accessed here:

http://www.biomedcentral.com/1471-2458/14/431/prepub
